# Use of GeneXpert and the role of an expert panel in improving clinical diagnosis of smear-negative tuberculosis cases

**DOI:** 10.1371/journal.pone.0227093

**Published:** 2019-12-30

**Authors:** Jovilia Abong, Victoria Dalay, Ivor Langley, Ewan Tomeny, Danaida Marcelo, Victor Mendoza, Arvin Christian Aquino, Anna Marie Celina Garfin, Bertie Squire, Charles Yu

**Affiliations:** 1 Research Division, De La Salle Medical and Health Sciences Institute, Dasmariñas City, Cavite, Philippines; 2 Collaboration for Applied Health Research & Delivery, Liverpool School of Tropical Medicine, Liverpool, United Kingdom; 3 National TB Control Program, Department of Health, Manila, Metro Manila, Philippines; The University of Georgia, UNITED STATES

## Abstract

**Setting:**

A high proportion of notified tuberculosis cases in the Philippines are clinically diagnosed (63%) as opposed to bacteriologically confirmed. Better understanding of this phenomenon is required to improve tuberculosis control.

**Objectives:**

To determine the percentage of smear negative presumptive tuberculosis patients that would be diagnosed by GeneXpert; compare clinical characteristics of patients diagnosed as tuberculosis cases; and review the impact that the current single government physician and a reconstituted Tuberculosis Diagnostic committee (expert panel) may have on tuberculosis over-diagnosis.

**Design:**

This a cross-sectional study of 152 patients 15–85 years old with two negative Direct Sputum Smear Microscopy results, with abnormal chest X-ray who underwent GeneXpert testing and review by an expert panel.

**Results:**

Thirty-two percent (48/152) of the sample were Xpert positive and 93% (97/104) of GeneXpert negatives were clinically diagnosed by a single physician. Typical symptoms and X-ray findings were higher in bacteriologically confirmed tuberculosis. When compared to the GeneXpert results the Expert panel’s sensitivity for active tuberculosis was high (97.5%, 39/40), specificity was low (40.2%, 35/87).

**Conclusion:**

Using the GeneXpert would increase the level of bacteriologically confirmed tuberculosis substantially among presumptive tuberculosis. An expert panel will greatly reduce over-diagnosis usually seen when a decision is made by a single physician.

## Introduction

The Philippines ranks as 4^th^ globally in tuberculosis (TB) incidence among the high-burden countries, and 5^th^ among MDR-TB [1]. In 2016, the estimate of TB incidence among Filipino adults was 554 per 100,000 (95% C.I. 311─866) [2].

Local and international experts have noted the high level of TB cases that are Clinically Diagnosed (CD) (approximately 61% of notified cases in 2017) in the Philippines [2]. A better understanding of this phenomenon will play a major role in shifting the battle for TB control in the Philippines.

GeneXpert MTB/RIF (Xpert), introduced in the Philippines in 2011, is a rapid diagnostic test with high sensitivity and specificity for TB. Despite the availability of Xpert, direct sputum smear microscopy (DSSM) remains the primary diagnostic tool for pulmonary tuberculosis (PTB), although the national program is gradually shifting to Xpert.

Cavite, one of the largest provinces in the Philippines, has consistently low detection rates and high CD PTB [3,4]. Bacteriological confirmation by TB Culture from smear-negative patients is 7% however it is not routinely requested. Instead, smear negative pulmonary tuberculosis (SNPT) cases are referred to a single government physician for further evaluation and treatment recommendation.

The TB Diagnostic Committee (TBDC), composed of a local panel of experts from private and public sectors, was created in the 1990’s by the Department of Health (DOH) to improve the quality of diagnosis of sputum smear-negative cases using chest X-ray findings of patients from the TB Directly Observed Treatment (DOTS) Facility. The shortage of trained and qualified specialists especially in rural and remote areas in the Philippines however resulted in a longer turnaround time and delays in diagnosis and treatment. This led to a change in the Philippine Manual of Procedures which then gave an option of a single physician (SP) making a decision instead of a TBDC. This may have contributed to the steady rise of clinical diagnosis in recent years. Oftentimes an SP (sometimes acting as a ‘one-man TBDC’) can decide on diagnosis and treatment of clinical TB but some TBDCs continue to function [5].

This study is part of a larger project called the Newton Agham Fund Impact assessment of diagnostic algorithms and tools for multi drug resistant (MDR-TB) and drug sensitive tuberculosis (TB) in the Philippines (TB-FIT). The primary aim of this study is to evaluate the role of GeneXpert in the diagnosis of BC TB among SNPT cases in a high CD prevalent area of Cavite.

## Methods

### Setting

The study was conducted in two urban-based public DOTS centers in the province of Cavite (Dasmariñas—659,019 population, Trece Martires City -155,713 population)[6]. These two facilities were chosen due to the high volume of patients and accessibility of Xpert testing. The study was conducted with the approval of the Provincial Health Office and the City Health Offices (CHO) of the two sites.

### Eligible patients

Patients seeking consult at Dasmariñas City and Trece Martires CHOs DOTS facilities with two negative sputum smears and with abnormal chest X-ray, were recruited into the study.

### Procedures

Direct Sputum Smear Microscopy was performed using Ziehl-Neelsen microscopes. Patients without chest X-rays or without film available, were requested to obtain a chest X-ray at the CHO/De La Salle. Eligible patients were offered the Xpert test and instructed on the test evaluation and sputum collection procedure.

Field coordinators secured informed consent and interviewed the eligible patients. A pre-tested tool was used to collect baseline data on demographics, clinical symptoms and chest X-ray. X-ray films were reproduced using a 1080 x 1920 resolution and 18 megapixels camera. X-ray results were classified based on standard radiologic reading of chest films utilized by certified local radiologic society (Philippine College of Radiology) [7–9]. All Xpert tests were performed in De La Salle Medical and Health Sciences Institute—Center for TB Research by the National TB Reference Laboratory trained medical technologist with a 100% Data Quality Check performance (conducted every quarter). Patients were instructed on sputum collection according to the National TB Program Manual of Procedures [5], sputum samples of patients were collected every Tuesday and Wednesday for Trece Martires CHO and daily for Dasmariñas CHO and stored at -20°C until testing.

Xpert testing was performed according to the manufacturer’s procedure. Those who were Xpert negative were referred to the corresponding SP of the participating sites for final disposition. Patients recommended for treatment by the SP, were considered as CD TB. In addition, a panel of experts consisting of 3 certified pulmonologists, a microbiologist and a radiologist reviewed the clinical profile and chest X-rays of all study participants. The panel was unaware of the Xpert results prior to their evaluations. If there was a disagreement with the diagnosis among the members, consensus of the majority (at least 3 of 5 panel members) was needed to carry out the diagnostic recommendation.

Physicians were provided with the results of the Xpert prior to patient’s consult. Treatment statuses of the patients were obtained within a month after notification of Xpert result.

### Sample size calculation

A sample size of 152 SNPT patients were included in the study to be able to have 95% confidence level of estimating percentage of Xpert positivity at 21.3% [10] with relative precision of 30%, participation rate of 85%. The computed sample size was proportionately allocated to Dasmariñas CHO 1 and Trece Martires CHO using 2015 data on number of smear negatives from the two sites.

### Data analysis

The survey was paper-based and EPI Info for Windows v7.2 was used for data entry and analysis. A Chi-square test was used to determine significant differences in categorical variables such as demographic and clinical characteristics between those Xpert positive and CD patients, while a t-test was used to determine significant differences in continuous variables such as age and number of symptoms. Level of significance was set at p-value less than 0.05.

### Ethics statement

This study was approved by the Independent Ethics Committee of the De La Salle Medical and Health Sciences Institute with the approval number of–DLSHSI-IEC (2017)– 02 -01- A. All participants were provided a written informed consent.

## Results

From June to August 2017, we recruited 152 patients who fulfilled the study’s inclusion criteria. They were all smear-negative participants with an abnormal chest X-ray, and all consented to participate and be tested with Xpert. The mean age was 40.3 years (range 15–85 years); 62.7% were male.

Thirty-two percent (48/152, 95% CI 24.6, 39.3) tested positive on Xpert and were enrolled in treatment at their corresponding DOTS facility. An average of 3 smear-negative presumptive PTB patients needed to be Xpert tested to detect 1 Bacteriologically Confirmed (BC) PTB. One rifampicin resistant case was detected and enrolled at the *Programmatic Management of Drug-resistant TB Centre* (PMDT) for treatment.

**Fig 1:**
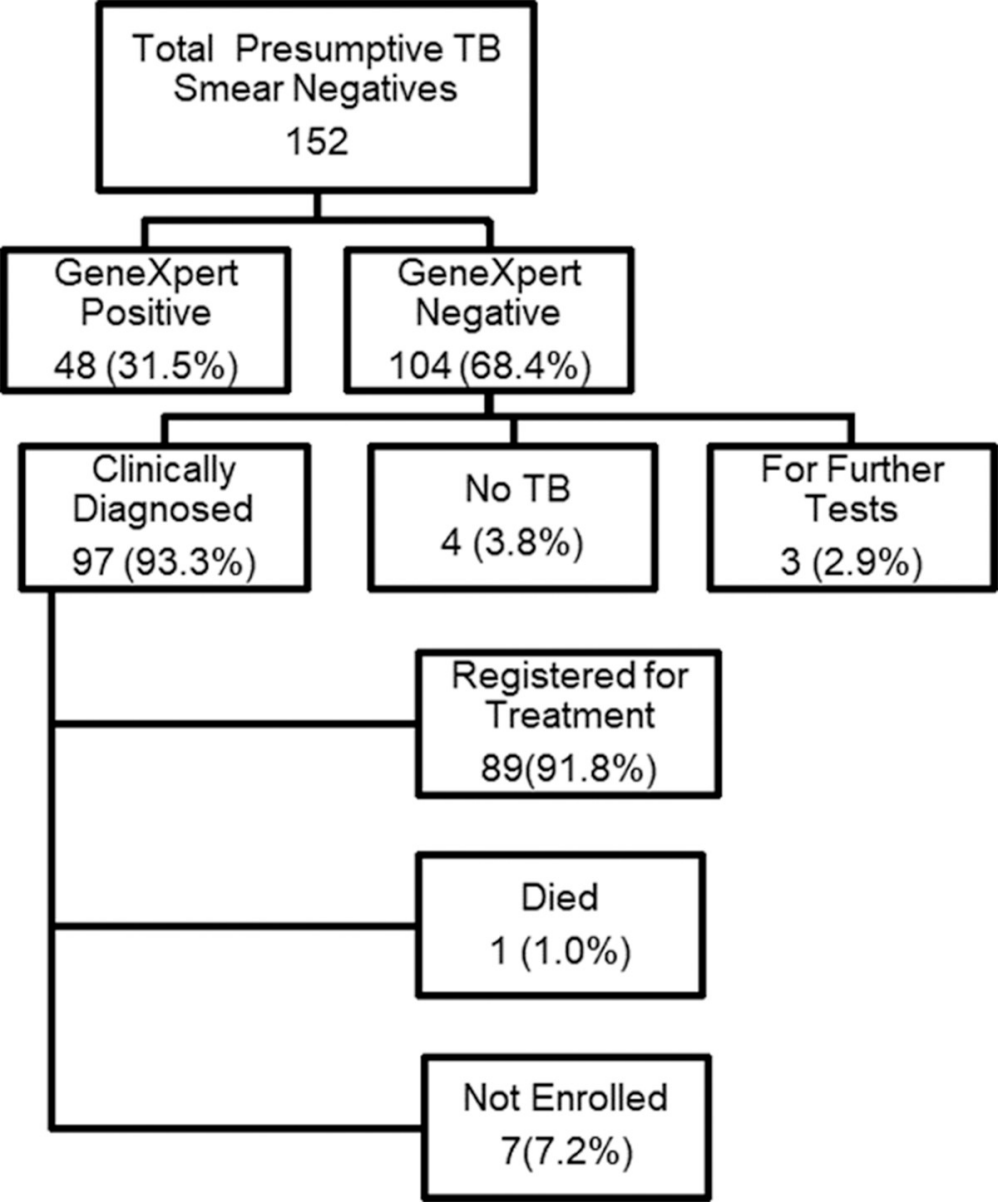
Diagnostic outcome of 152 smear negative presumptive TB patients.

Ninety-seven of the 104 (93.3%) smear-negative, Xpert negative patients were CD by SP and were enrolled for treatment. Of the remaining seven, four were diagnosed with no PTB, three patients needed further diagnostic tests (repeat chest X-ray, chest CT scan. These three were lost to follow-up (LTFU). Only 89 CD patients were enrolled to treatment (Fig 1). The remaining 8 patients were not enrolled due to refusal to treatment (1 patient), diagnosed with no active TB (2 patients), LTFU (4 patients) and 1 died of a non-TB related heart condition (Fig 1).

Tables 1–4 show the demographic profile, reason for consultation, Body Mass Index and symptoms of the 145 smear-negative TB patients, (48 Xpert positive and 97 clinically diagnosed cases).

**Table 1:** Demographic profile of bacteriologically confirmed through Xpert (BC) and clinically diagnosed (CD) among smear negative presumptive TB patients in TB DOTS facility, Dasmariñas and Trece Martirez City, 2017.

*Characteristics*		*BC (n = 48)*		*CD (n = 97)*		*Chi-Square* *p-value*
		n	%	n	%	
*Sex*	Female	14	29.2	40	41.2	0.157
	Male	34	70.8	57	58.8	
*Age Group*	< 45 yo	36	75	57	58.8	0.055
	= >45 yo	12	25	40	41.2	
*Mean (SD) Age in years*		36.8	(13.8)	40.9	(18.6)	0.132
*Employment*	Yes	21	43.8	37	38.1	0.517
	No	27	56.3	60	61.9	
*Educational Attainment*	Not attended school / Primary School	14	29.2	32	33	0.642
	Secondary school / University/ College	34	70.8	65	67	
*Body Mass Index Category*	Underweight	27	56.3	28	28.9	0.009
	Normal	19	39.6	55	56.7	
	Overweight	1	2.1	11	11.3	
	Obese	1	2.1	3	3.1	
*Smoking*	Current smoker	12	26.1	27	28.4	0.476
	Former smoker	5	100	5	5.3	
	Never smoked	29	63	63	66.3	
*Alcohol*	Drinker	28	58.3	54	55.7	0.761
	Non-drinker	20	41.7	43	44.3	

**Table 2:** Diagnosis outcome of subjects who consulted for employment or check-up among smear negative presumptive TB patients in TB DOTS facility, Dasmariñas and Trece Martirez City, 2017.

Reason for Consultation	Diagnosis Outcome					
	BC		CD		Total	
	n	%	n	%	n	%
For employment	2	8.3	22	91.7	24	100
Check-up	46	38.0	75	62.0	121	100
Total	48	33.1	97	66.9	145	100

**Table 3:** Symptoms of bacteriologically confirmed through Xpert (BC = 48) and clinically diagnosed (CD = 97) among smear negative presumptive TB patients in TB DOTS facility, Dasmariñas and Trece Martirez City, 2017.

TB Symptoms	BC (n = 48)		CD (n = 97)		OR	p-value
	n	%	n	%	(95% CI)	
Cough	43	90	64	66	4.4 (1.6, 12.3)	0.002
Weight loss	31	65	31	32	3.9 (1.9, 8.1)	<0.001
Hemoptysis	12	25	8	8	3.7 (1.4, 9.8)	0.006
Night sweats	18	38	15	16	3.3 (1.5, 7.3)	0.003
Fever	20	42	18	19	3.1 (1.5, 6.8)	0.003
Loss of appetite	24	50	28	29	2.5 (1.2, 5.0)	0.013
Malaise	16	33	16	16	2.5 (1.1, 5.7)	0.021
Shortness of breath/chest pain	31	65	42	43	2.4 (1.2, 4.9)	0.016
Sputum Production	40	83	68	70	2.1 (0.9, 5.1)	0.085
No of symptoms Mean (SD)	5	(2.3)	3	(2.2)	--	<0.001

**Table 4:** Chest X-ray findings of bacteriologically confirmed through Xpert (BC = 48) and clinically diagnosed (CD = 97) among smear negative presumptive TB patients in TB DOTS facility, Dasmariñas and Trece Martirez City, 2017.

Chest X-ray findings	BC (n = 48)		CD (n = 97)		OR	*p*-value
	n	%	n	%	(95% CI)	
Extensive Involvement / no cavitations	18	38	5	5	11.0 (3.8, 32.3)	<0.001
Miliary TB	4	8	1	1	8.7 (0.9, 80.4)	0.023
Cavitation	15	31	5	5	8.4 (2.8, 24.8)	<0.001
Pleural effusion	12	25	4	4	7.8 (2.3, 25.6)	<0.001
Consolidation	20	42	11	11	5.6 (2.4, 13.1)	<0.001
Fibrohazed densities	45	94	72	74	5.2 (1.5, 18.3)	0.005
Opacities, densities	31	65	26	27	4.9 (2.4, 10.5)	<0.001
Suspected Pneumonia	12	25	11	11	2.6 (1.1, 6.4)	0.034
Hazy densities	32	67	50	52	1.9 (0.9, 3.9)	0.084
Fibrosis, pleural thickening	12	25	20	21	1.3 (0.6, 2.9)	0.549
Atelectasis	3	6	8	8	0.7 (0.2, 2.9)	0.669
Calcification	0	0	3	3	--	--

Table 1 shows that the baseline characteristics of the two groups were similar except for the Body Mass Index. A significant number of patients with positive Xpert results exhibited abnormal Body Mass Index, more than half were underweight.

In Table 2 a significantly higher proportion of patients who went to the DOTS clinic for employment reasons were CD, compared to those who consulted for check-up (91.7% vs 62%) (*p*-value = 0.004).

All BC patients were symptomatic with an average of 5 symptoms while the CD presented an average of 3 (*p*-value <0.001). Prevalence of the following symptoms were higher among the BC patients than CD: cough (p-value = 0.002), loss of appetite (p-value = 0.013), weight loss (p-value <0.001), shortness of breath (p-value = 0.016). Cough, weight loss, and hemoptysis were 4 times (3.7 to 4.4. OR) more likely in BC than in CD (Table 3).

Among the Xpert negative CD patients the most common symptoms included cough and shortness of breath/chest pain (Table 3). 15 (15.4%) CD patients were asymptomatic, 8 consulted for employment purposes, 2 were referred by private physicians, and 5 were walk-ins.

Chest X-rays of all patients were reviewed by the panel of experts. Radiologic findings of extensive involvement, miliary TB, cavitation, pleural effusion, consolidation, fibrohazed, opacities are significantly higher among BCs compared to CDs (p values <0.05) (Table 4)

Table 5 shows the Expert panel’s (EP) diagnosis compared to Xpert positivity of 127 presumptive smear negative patients. Twenty-five patients recommended for repeat chest X-ray by the EP were not included in the computation.

**Table 5:** Diagnosis of expert panel and Xpert results of smear negative presumptive TB patients in TB DOTS facility, Dasmariñas and Trece Martirez City, 2017.

Expert Panel (EP)	Xpert Result					
	MTB Detected		MTB Not Detected		Total	
	n	%	n	%	n	%
Active TB / Indeterminate	39	81	52	50	91	60
Inactive TB / No TB / Other lung disease	1	2	35	34	36	24
For Further Test	8	17	17	16	25	16
Total	48	100	104	100	152	100

Excluding those for further testing, the EP diagnosed active PTB in 39 of 40 Xpert positive patients yielding a 98% sensitivity (based on Xpert as the gold standard), and further diagnosed 35 out of 87 Xpert negatives as non-active TB yielding a 40% specificity.

Of the 104 Xpert negative cases, the EP considered 52 cases, 28 of which were active PTB cases and 24 indeterminate cases. According to the panel these 24 indeterminate cases should be enrolled for treatment because of the presumption that it is better to treat them than leave them untreated since some may really have active TB. Yousang Ko et al. in 2018 evaluated the diagnostic performance of the radiographic CT scan activities in predicting definite and overall PTB in patients with presumed PTB and found that 10% of indeterminate PTB were culture confirmed [11].

Table 6 shows comparison between an SP and EP’s diagnosis. Excluding 20 subjects that were recommended for further test by either the SP or EP, among the 84 smear and Xpert negative patients with abnormal X-ray, 80 (95%) were CD by a single physician of whom only 51 (61%) were considered CD by the EP.

**Table 6:** Comparison of diagnosis of expert panel vs a single physician diagnosis of presumptive smear negative patients among smear negative presumptive TB patients in TB DOTS facility, Dasmariñas and Trece Martirez City, 2017.

Single Physician (SP)	Expert Panel (EP)							
	Active TB / Indeterminate		Inactive TB / No TB / Other lung disease		For Further Test		Total	
	n	%	n	%	n	%	n	%
CD	51	98	29	83	17	100	97	93
No TB	0	0	4	11	0	0	4	4
For Further Test	1	2	2	6	0	0	3	3
Total	52	100	35	100	17	100	104	100

## Discussion

### Xpert positivity among smear negative presumptive tuberculosis

Across two DOTS centers in Cavite, testing SNPT cases with GeneXpert returned a bacteriological confirmation in 31% of cases (i.e. we only need to test 3 smear negative cases to detect 1 BC). This percentage is consistent with the range of figures from other studies (21% - 36.5%).

In a fee paying hospital in Nepal 21% of smear negative cases were bacteriologically confirmed by Xpert and the number needed to test was five [10]. A higher percentage (34.9%) of smear negatives with positive Xpert result was reported in a hospital-based setting in India, needing 2.8 to be tested to detect one positive Xpert SNPT [12]. Among culture-positive, smear-negative patients, Lombardi reported an increased TB detection of 36.5% by Xpert [13].

### Comparison of clinical and radiologic features of BCs and CDs

Known clinical predictors of PTB such as cough, weight loss, hemoptysis and classic radiologic findings seen on x-ray, were observed among many Xpert positive cases as expected [14,15]. There was a significant percentage of smear and Xpert negative cases who were diagnosed to have SNPT and were enrolled to treatment.

Three features may have contributed to the high SNPT. Firstly, clinical diagnosis of SNPT relied on chest X-ray findings as demonstrated among fifteen asymptomatic smear and Xpert negative cases who were diagnosed based on chest X-ray findings of apical infiltrates/densities. Secondly, the study inclusion criteria required an abnormal chest X-ray, which increased the likelihood of being CD, reaching 93% of all smear and Xpert negative subjects diagnosed as CD by the SP. Thirdly, there was a high percentage of CD cases among subjects whose consultation was for employment purposes.

### Single physician versus expert panel

Based on the NTP Manual of Procedures, the TBDC or a Physician may diagnose patients as CD. This was introduced in 2014 due to slow turnaround times of the TBDC decisions which delayed treatment [16]. While SP may hasten the process of decision-making avoiding potential pitfalls of delays of convening a panel, we found that this may also be contributing to over-diagnosis and high treatment enrolment. On the “patient pressure” side, local efforts are being made to harmonize employment policies through inter-agency coordination (however this does not apply to foreign based employment). The 5-member EP created for the study to simulate a fully functioning TBDC evaluated all the participants’ chest X-ray films and their clinical history. To address some of the challenges facing the present TBDC, establishing an eTBDC (electronic TBDC) that doesn’t need face to face meeting may be a good alternative to reduce turn-around time and unnecessary delays. The eTBDC could meet through web conferencing at a time convenient to the members which would eliminate travel time and cost. Through this method, detailed information about symptomatology and X-ray finding which, in this study, were significantly found to be present in Xpert positive patients will help toward a more accurate diagnosis of TB. The eTBDC has been studied and piloted in the Philippines and was found to have great potential [17].

Using a published sensitivity of 67% and specificity of 98% [18] of Xpert among the 152 smear-negative cases for which we have results, two patients would be estimated to be unnecessarily treated (false positives). The estimated number of patients with TB disease that would be missed by Xpert in the 152 population is 23. Although it’s not possible in this analysis to determine the actual TB status of individual patients it is interesting to note the EP identified 28 additional active PTB cases which is not dissimilar to the 23 calculated above and suggests the EP was able to identify most of these cases without generating high levels of over diagnosis.

The higher proportion of CD patients among those attending pre-employment ‘fit to work’ checks supports the hypothesis that there are non-medical factors leading to physicians enrolling patients onto TB treatment. The authors believe the high CD rate among those subjects is largely due to patient-pressure, with patients seeking to be placed on treatment as a way of being cleared as fit to work. The study sites are industrialized areas in Cavite where several industrial zones are located, and similarly in surrounding municipalities. Chest X-ray is a mandatory pre-employment procedure [5], and those with suspicious chest X-ray results are referred to physician specialists or DOTS centers. Patients with indeterminate findings are typically advised to get a repeat X-ray after 3–6 months, delaying medical clearance by issuance of a ‘fit to work’ certificate. In this case this delay would cost the applicant their job position, greatly impacting both the patient and their family, resulting in both economic loss and potentially long lasting mental and physical health implications. These considerations likely pose a serious challenge to TB DOTS physicians/TBDCs when diagnosing such patients.

Over-diagnosis leads to additional health system and direct patient costs. Patients incur costs because of repeated visits to health centers, and exposure to potentially serious side effects from unnecessary drugs. Patients misdiagnosed to have TB may actually have another serious illness–e.g. cancer that requires immediate attention.

In this study an SP made decisions on clinical diagnosis. A fully represented TBDC exemplified by the EP, may be able to diagnose correctly those with active TB (SNPT) missed by Xpert. This study suggests that a fully represented TBDC is still useful to address missed cases not identified by available diagnostic tests including Xpert and should be the preferred option in diagnosing SNPT.

Recently, the WHO [19] released a consolidated summary of recommendations for the use of X-ray for the diagnosis of TB advocating its widespread use in increasing detection rates for TB in high burden countries. The widespread use of X-ray for screening and improving the case detection of TB is being strongly recommended in the Philippines. However, this study has shown that the poor quality of many of the X-rays in the field, both public and private, in industrial and small clinics and hospitals are a major barrier to making an accurate diagnosis. Other issues include inter-observer variability and pressures on physicians to treat among other challenges. A shift to digital X-rays will go a long way in addressing quality issues, while further training of physicians in the proper interpretation of X-rays for TB diagnosis will help high burden TB countries. A stricter quality assurance should be implemented for use of routine X-rays.

## Limitations

The X-rays obtained in the two sites in both public and private were mostly non-digital, many were of poor quality. The use of Xpert as the gold standard instead of culture may have missed some cases because of its lower sensitivity. There may be truly CD cases that are both smear-negative and Xpert negative. It must also be pointed out however that the EP in this study may not truly represent TBDCs nationwide.

## Conclusion

The impact of Xpert on the diagnosis of confirmed TB is substantial, identifying potentially 31% of smear negative presumptive cases as BC PTB. Clinical and X-ray features among Xpert positive cases supported the accuracy of Xpert. The EP is likely to have high sensitivity but low specificity for diagnosing presumptive smear-negative tuberculosis when compared to the Xpert results. The number of patients enrolled to treatment was not influenced by Xpert results. There was still a high percentage of smear-negative, Xpert negative cases who were enrolled to treatment upon the decision of an SP. A combination of factors such as relying on chest X-ray for diagnosis of SNPT, inclusion criteria of abnormal chest X-ray, implication of opportunity cost among patients seeking pre-employment clearance contributed significantly to the high percentage of clinically diagnosed SNPT.
